# A real−world pharmacovigilance study of FDA Adverse Event Reporting System events for pralsetinib

**DOI:** 10.3389/fonc.2024.1491167

**Published:** 2024-11-12

**Authors:** Yi Yin, Fengli Sun, Youpeng Jin

**Affiliations:** ^1^ Department of Pediatric Intensive Care Unit, Shandong Provincial Hospital Affiliated to Shandong First Medical University, Jinan, Shandong, China; ^2^ Department of Pediatric Intensive Care Unit, The Second Hospital, Cheeloo College of Medicine, Shandong University, Jinan, Shandong, China

**Keywords:** FDA Adverse Event Reporting System (FAERS), pralsetinib, pharmacovigilance, real-world analysis, adverse event (AE)

## Abstract

**Background:**

Pralsetinib, a selective oral inhibitor of rearranged during transfection (RET) fusion proteins and oncogenic RET mutants, has shown significant efficacy in treating RET fusion-positive non-small cell lung cancer and thyroid cancer. However, since pralsetinib was approved in the United States in September 2020, there have been limited reports of post-marketing adverse events (AEs). In this study, we aimed to analyze the AE signals with pralsetinib on the basis of the United States Food and Drug Administration (FDA) Adverse Event Reporting System (FAERS) to provide instructions in clinical practice.

**Methods:**

All AE reports were obtained from the FAERS database from the first quarter (Q3) of 2020 to the second quarter (Q2) of 2024. Various signal quantification techniques were used for analysis, including reporting odds ratios, proportional reporting ratios, Bayesian confidence propagation neural network, and multi-item gamma Poisson shrinker (MGPS)-based empirical Bayesian geometric mean.

**Results:**

Out of 8,341,673 case reports in the FAERS database, 1,064 reports of pralsetinib as the “primary suspected (PS)” AEs were recorded, covering 26 system organ classes and 256 preferred terms. Of the reports, 62.5% were from consumers rather than healthcare professionals. The most common systems were general disorders and administration site conditions (n = 704), investigations (n = 516), and gastrointestinal disorders (n = 405). A total of 95 significant disproportionality preferred terms (PTs) conformed to the four algorithms simultaneously. AEs that ranked the top three at the PT level were hypertension (n = 80), asthenia (n = 79), and anemia (n = 65). Of the 95 PTs with significant disproportionation, unexpected significant AEs such as increased blood calcitonin, increased myocardial necrosis marker, and bacterial cystitis were observed, which were not mentioned in the drug’s instructions. The median onset time of pralsetinib-associated AEs was 41 days [interquartile range (IQR) 14–86 days]. The majority of the AEs occurred in 30 days (42.86%).

**Conclusion:**

Our pharmacovigilance analysis of real-world data from the FEARS database revealed the safety signals and potential risks of pralsetinib usage. These results can provide valuable evidence for further clinical application of pralsetinib and are important in enhancing clinical medication safety.

## 1 Introduction

Rearranged during transfection (RET) gene fusions are oncogenic events detected in 1%–2% of non-small cell lung cancer (1), 20% of papillary thyroid carcinomas (2), and less frequently in other tumor types (3).

Pralsetinib is a potent and selective oral inhibitor of RET fusion proteins and oncogenic RET mutants (4). It is approved in the United States for the treatment of adult patients with metastatic RET fusion-positive non-small cell lung cancer (NSCLC) and adults and pediatric patients 12 years and older with advanced or metastatic RET fusion-positive thyroid cancer who require systemic therapy and who are radioactive iodine-refractory.

Clinical trials have highlighted the effectiveness of pralsetinib in non-small cell lung cancer and thyroid cancer (5–7). Although these clinical trials have clarified common adverse events such as neutropenia, hypertension, and elevated aminotransferase, ongoing post-marketing monitoring is critical owing to the widespread use of pralsetinib. In view of the complexity of adverse event reporting and the potential for underreporting in clinical trials, real-world databases such as the Food and Drug Administration (FDA) Adverse Event Reporting System (FAERS) play a key role in enabling researchers to fully analyze post-approval safety data (8). By analyzing the FAERS data in depth, we can obtain a more comprehensive understanding of the safety profile and adverse reaction characteristics of pralsetinib. In this study, we aimed to analyze the AE signals with pralsetinib on the basis of the United States FAERS to provide instructions in clinical practice.

## 2 Methods

### 2.1 Study design and data sources

To assess the post-market safety of pralsetinib, we conducted a retrospective pharmacovigilance study using data obtained from the FAERS database, spanning from the first quarter (Q3) of 2020 to the second quarter (Q2) of 2024. The FAERS data files consist of seven databases, namely, demographic and administrative information (DEMO), adverse drug reaction information (REAC), patient outcome information (OUTC), drug information (DRUG), drug therapy start and end dates (THER), information on report sources (RPSR), and indications for use/diagnosis (INDI). According to FDA guidelines, we removed duplicate reports. We selected the latest FDA_DT for the same CASEID or chose the higher PRIMARYID in cases where both the CASE number and FDA_DT were identical.

**Figure 1:**
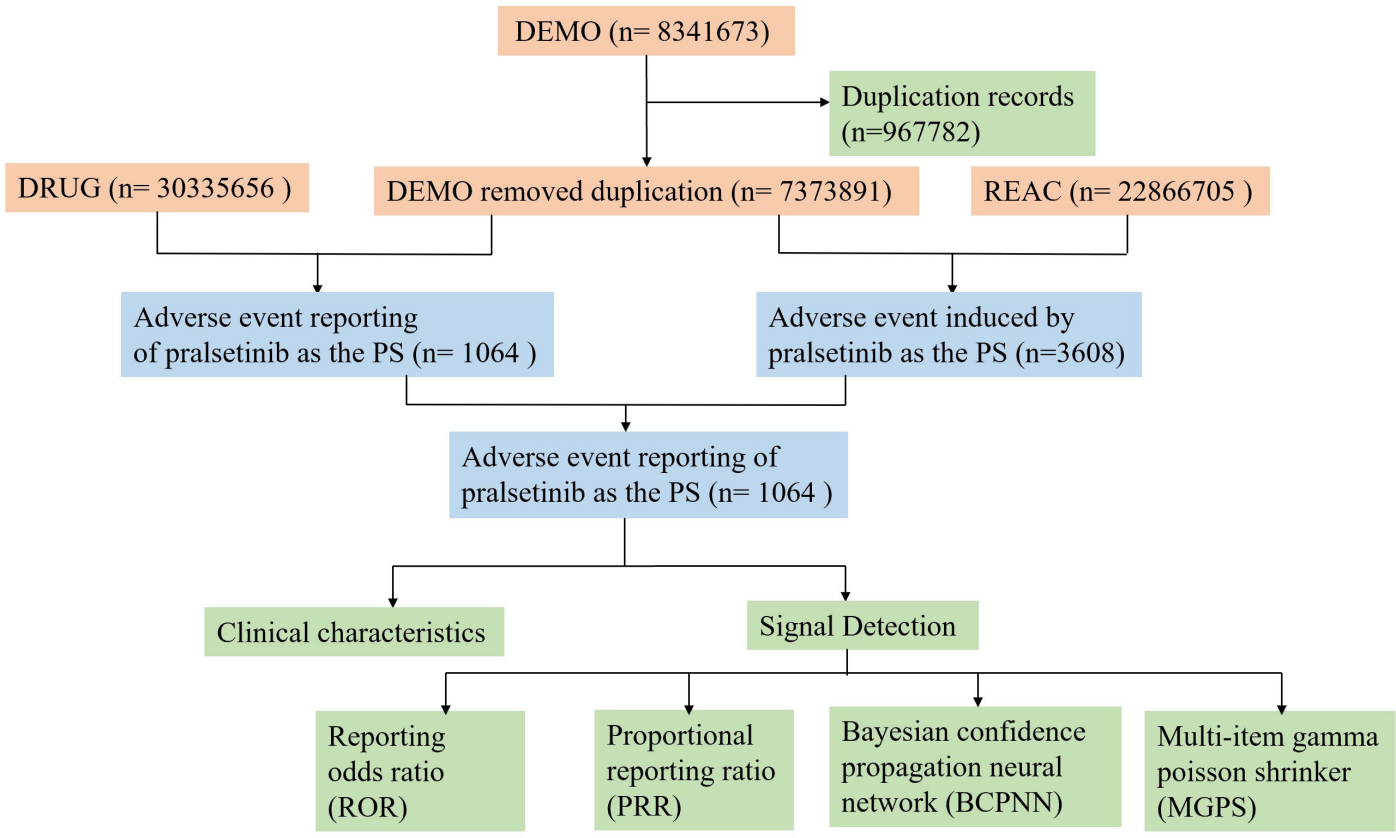
The flow diagram of selecting pralsetinib-related AEs from FAERS database.

Adverse events (AEs) associated with pralsetinib were extracted from the REAC dataset. Preferred terms (PTs) with reporting counts of ≥3 were selected. All PTs in the REAC dataset were classified into the corresponding primary system organ class (SOC) according to the standardized Medical Dictionary for Regulatory Activities (MedDRA) version 26. Only AEs where pralsetinib was identified as the primary suspected (PS) drug were included in the analysis (**Figure 1**).

### 2.2 Statistical analysis

Disproportionality analysis was considered a pivotal tool for evaluating potential associations between specific AEs and particular drugs (9). Four statistical procedures were applied: reporting odds ratio (ROR), proportional reporting ratio (PRR), Bayesian confidence propagation neural network (BCPNN), and multi-item gamma Poisson shrinker (MGPS)-based empirical Bayesian geometric mean (EBGM) (10). Specific formulas and thresholds are outlined in **Supplementary Table S1**. Statistical analysis was conducted using R_4.4.1 software.

## 3 Results

### 3.1 Descriptive analysis

The results showed that a total of 8,341,673 adverse events were collected in the FAERS database spanning from the first quarter (Q3) of 2020 to the second quarter (Q2) of 2024. Among these, 1,064 AE reports identified pralsetinib as the PS drug. In terms of gender, there were 525 female cases (49.3%), 401 male cases (37.7%), and 138 cases (13%) with partial gender information missing. The main reporting countries were the United States (50.8%) and China (31.7%). In addition, the majority of reports (62.5%) were from consumers rather than healthcare professionals.

Lung neoplasm malignant was the most reported indication (37.5%), followed by non-small cell lung cancer (18.8%), product used for unknown indication (11.9%), and thyroid cancer (11.7%). In terms of clinical outcomes, apart from the unknown adverse events, those leading to hospitalization or prolongation of hospitalization were most frequent (25.0%), followed by other serious events (20.0%) or death (15.6%). The details can be found in **Table 1**.

**Table 1:** Clinical characteristics of reports with pralsetinib from the FAERS database (January 2004 to June 2024).

Characteristics	Case number, n	Case proportion, %
Year of report		
2020	33	3.1
2021	434	40.8
2022	270	25.4
2023	199	18.7
2024	128	12.0
Sex		
Female	525	49.3
Male	401	37.7
Unknown	138	13.0
Age (years)		
<18	2	0.2
18–64.9	302	28.4
65–85	300	28.2
>85	5	0.5
Unknown	455	42.8
Weight (kg)		
<50	14	1.3
50–100	117	11.0
>100	12	1.1
Unknown	455	42.8
Reported countries (top 5)		
USA	540	50.8
China	337	31.7
France	40	3.8
Italy	30	2.8
Korea	12	1.1
Reporter		
Physician	238	22.4
Health professional	112	10.5
Pharmacist	45	4.2
Not reported	4	0.4
Consumer	665	62.5
Indications (top 5)		
Lung neoplasm malignant	399	37.5
Non-small cell lung cancer	200	18.8
Product used for unknown indication	127	11.9
Thyroid cancer	125	11.7
Medullary thyroid cancer	35	3.3
Outcomes		
Death	166	15.6
Disability	3	0.3
Hospitalization	266	25.0
Life-threatening	11	1.0
Other serious events	213	20.0
Unknown	405	38.1

FAERS, Food and Drug Administration Adverse Event Reporting System.

### 3.2 Signal detection of pralsetinib at the system organ class level

**Figure 2:**
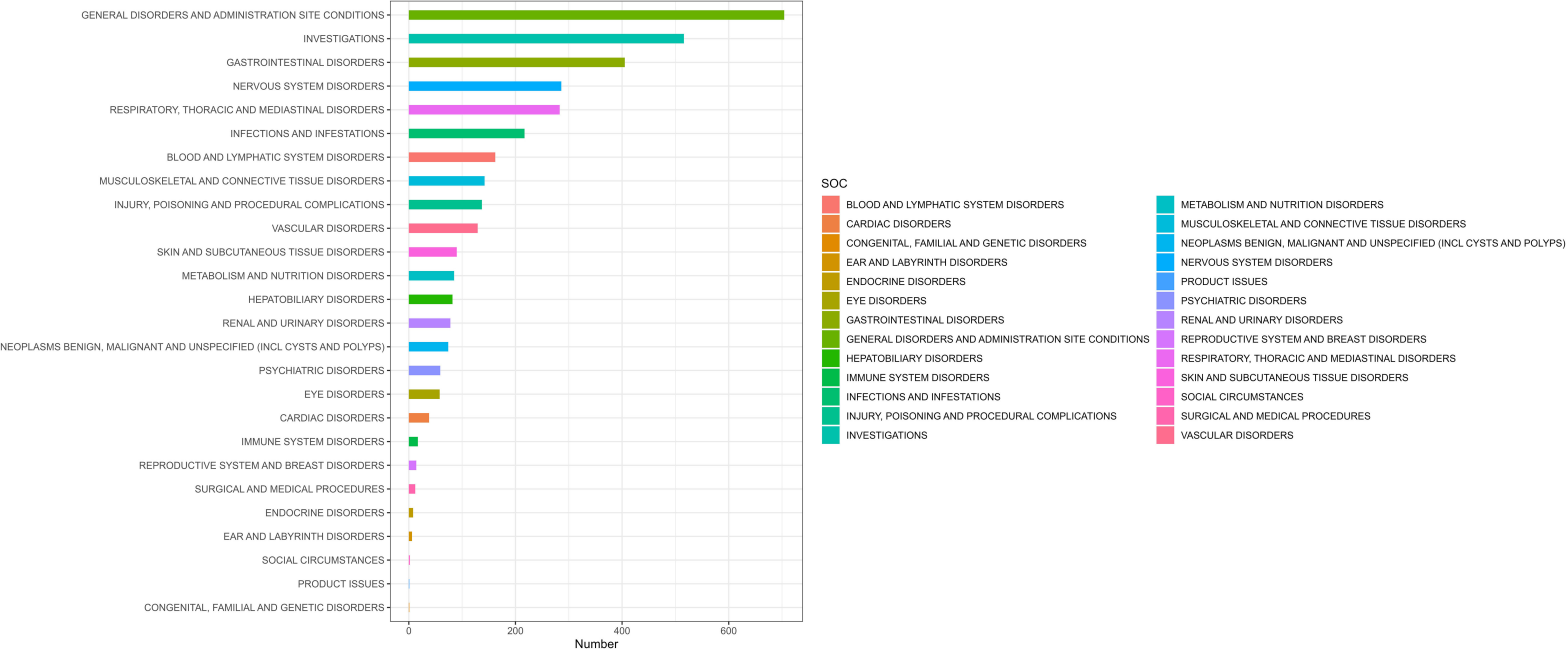
The number of AEs induced by pralsetinib at the SOC level.

The number of AEs induced by pralsetinib as the “PS” at the SOC level is shown in **Figure 2**. The study indicates that 3,608 adverse event reports (AERs) induced by pralsetinib occurred across 26 organ systems. The most common systems were general disorders and administration site conditions (n = 704, ROR 1.13, PRR 1.11, IC 0.14, and EBGM 1.11), investigations (n = 516, ROR 2.65, PRR 2.42, IC 1.27, and EBGM 2.42), and gastrointestinal disorders (n = 405, ROR 1.48, PRR 1.42, IC 0.51, and EBGM 1.42). The details can be found in **Table 2**.

**Table 2:** Signal strength of AEs of pralsetinib at the SOC level in FAERS database.

System organ class (SOC)	N	ROR (95% CI)	PRR (χ^2^)	IC (IC025)	EBGM (EBGM05)
General disorders and administration site conditions	704	1.13 (1.04–1.23)	1.11 (8.57)	0.14 (−1.52)	1.11 (1.03)
Investigations	516	2.65 (2.42–2.91)	2.42 (454.82)	1.27 (−0.39)	2.42 (2.23)
Gastrointestinal disorders	405	1.48 (1.33–1.64)	1.42 (55.16)	0.51 (−1.16)	1.42 (1.3)
Nervous system disorders	286	1.1 (0.97–1.24)	1.09 (2.33)	0.13 (−1.54)	1.09 (0.99)
Respiratory, thoracic, and mediastinal disorders	283	1.78 (1.58–2.01)	1.72 (89.16)	0.78 (−0.89)	1.72 (1.55)
Infections and infestations	217	1.04 (0.91–1.2)	1.04 (0.36)	0.06 (−1.61)	1.04 (0.93)
Blood and lymphatic system disorders	162	2.73 (2.33–3.19)	2.65 (169.4)	1.41 (−0.26)	2.65 (2.32)
Musculoskeletal and connective tissue disorders	142	0.74 (0.63–0.88)	0.75 (12.1)	−0.41 (−2.08)	0.75 (0.65)
Injury, poisoning, and procedural complications	137	0.28 (0.24–0.33)	0.31 (241.81)	−1.7 (−3.36)	0.31 (0.27)
Vascular disorders	129	1.97 (1.65–2.35)	1.93 (59.23)	0.95 (−0.72)	1.93 (1.67)
Skin and subcutaneous tissue disorders	90	0.45 (0.36–0.55)	0.46 (59.73)	−1.12 (−2.78)	0.46 (0.39)
Metabolism and nutrition disorders	85	1.24 (1–1.53)	1.23 (3.78)	0.3 (−1.37)	1.23 (1.03)
Hepatobiliary disorders	82	2.82 (2.27–3.51)	2.78 (94.2)	1.47 (−0.19)	2.78 (2.31)
Renal and urinary disorders	78	1.18 (0.94–1.47)	1.17 (2.03)	0.23 (−1.44)	1.17 (0.97)
Neoplasm benign, malignant, and unspecified (incl cysts and polyps)	74	0.53 (0.42–0.67)	0.54 (30.14)	−0.89 (−2.56)	0.54 (0.45)
Psychiatric disorders	59	0.29 (0.23–0.38)	0.31 (98.39)	−1.71 (−3.38)	0.31 (0.25)
Eye disorders	58	0.84 (0.65–1.09)	0.84 (1.81)	−0.25 (−1.92)	0.84 (0.68)
Cardiac disorders	38	0.54 (0.4–0.75)	0.55 (14.34)	−0.86 (−2.53)	0.55 (0.42)
Immune system disorders	17	0.41 (0.25–0.65)	0.41 (14.77)	−1.29 (−2.96)	0.41 (0.27)
Reproductive system and breast disorders	14	0.65 (0.39–1.11)	0.66 (2.56)	−0.61 (−2.28)	0.66 (0.42)
Surgical and medical procedures	12	0.22 (0.13–0.4)	0.23 (32.06)	−2.14 (−3.81)	0.23 (0.14)
Endocrine disorders	8	0.83 (0.41–1.65)	0.83 (0.29)	−0.28 (−1.94)	0.83 (0.46)
Ear and labyrinth disorders	6	0.4 (0.18–0.89)	0.4 (5.41)	−1.32 (−2.99)	0.4 (0.2)
Social circumstances	2	0.12 (0.03–0.46)	0.12 (13.6)	−3.11 (−4.78)	0.12 (0.04)
Product issues	1	0.01 (0–0.1)	0.01 (66.75)	−6.08 (−7.74)	0.01 (0)
Congenital, familial, and genetic disorders	1	0.1 (0.01–0.74)	0.1 (7.69)	−3.26 (−4.92)	0.1 (0.02)

AEs, adverse events; SOC, system organ class; FAERS, Food and Drug Administration Adverse Event Reporting System; ROR, reporting odds ratio; PRR, proportional reporting ratio; EBGM, empirical Bayesian geometric mean.

Notably, according to the ROR level, the top three SOCs with pralsetinib-associated AEs were hepatobiliary disorders [ROR 2.82 (2.27–3.51)], blood and lymphatic system disorders [ROR 2.73 (2.33–3.19)], and investigations [ROR 2.65 (2.42–2.91)], indicating a strong relationship with pralsetinib.

### 3.3 Signal detection of pralsetinib at the preferred term level

A total of 256 types of PTs were reported in different organ systems (**Supplementary Table S2**). We examined PT signals, with a total of 95 significant disproportionality PTs conforming to the four algorithms simultaneously (**Supplementary Table S3**). Among these, 59 PTs with significant disproportionation appeared in drug labels and clinical trial and post-marketing results, and we listed the top 20 PTS, as shown in **Table 3**; the top three AEs to pralsetinib, namely, hypertension (n = 80), asthenia (n = 79), and anemia (n = 65).

**Table 3:** The top 20 signal strength of adverse events that appear in the drug label or clinical trials/post-marketing experience of pralsetinib at the preferred term (PT) level that fit four algorithms simultaneously.

System organ class (SOC)	PTs	N	ROR (95% CI)	PRR (χ^2^)	IC (IC025)	EBGM (EBGM05)
Vascular disorders	Hypertension	80	6.87 (5.51–8.58)	6.74 (392.22)	2.75 (1.09)	6.74 (5.6)
General disorders and administration site conditions	Asthenia	79	4.06 (3.25–5.08)	4 (178.3)	2 (0.33)	3.99 (3.31)
Blood and lymphatic system disorders	Anemia	65	6.77 (5.3–8.66)	6.67 (313.83)	2.74 (1.07)	6.66 (5.43)
Investigations	White blood cell count decreased	63	9.01 (7.02–11.56)	8.87 (440.31)	3.15 (1.48)	8.86 (7.19)
General disorders and administration site conditions	Disease progression	60	8.44 (6.54–10.89)	8.31 (386.23)	3.05 (1.39)	8.3 (6.71)
Investigations	Blood pressure increased	58	6.18 (4.77–8.01)	6.1 (247.54)	2.61 (0.94)	6.09 (4.9)
Gastrointestinal disorders	Constipation	58	4.68 (3.61–6.06)	4.62 (164.92)	2.21 (0.54)	4.62 (3.72)
Investigations	Platelet count decreased	41	6.26 (4.6–8.52)	6.2 (179.02)	2.63 (0.96)	6.2 (4.79)
Hepatobiliary disorders	Hepatic function abnormal	37	18.23 (13.18–25.21)	18.05 (594.52)	4.17 (2.5)	18 (13.72)
Renal and urinary disorders	Renal impairment	34	6.61 (4.72–9.27)	6.56 (160.31)	2.71 (1.05)	6.56 (4.94)
Respiratory, thoracic, and mediastinal disorders	Pneumonitis	33	19.83 (14.07–27.96)	19.66 (582.95)	4.29 (2.63)	19.6 (14.71)
Gastrointestinal disorders	Dry mouth	30	7.61 (5.31–10.9)	7.55 (170.59)	2.92 (1.25)	7.55 (5.59)
Investigations	Hemoglobin decreased	30	5.68 (3.96–8.13)	5.64 (114.48)	2.49 (0.83)	5.63 (4.17)
Nervous system disorders	Hypoesthesia	25	3.25 (2.2–4.82)	3.24 (38.73)	1.69 (0.03)	3.24 (2.33)
Investigations	Blood creatinine increased	22	6.6 (4.34–10.03)	6.56 (103.69)	2.71 (1.05)	6.56 (4.62)
General disorders and administration site conditions	Edema peripheral	22	4.84 (3.18–7.36)	4.81 (66.48)	2.27 (0.6)	4.81 (3.39)
Respiratory, thoracic, and mediastinal disorders	Interstitial lung disease	19	6.81 (4.34–10.7)	6.78 (93.66)	2.76 (1.09)	6.78 (4.65)
General disorders and administration site conditions	Face edema	17	23.09 (14.33–37.22)	22.99 (356.33)	4.52 (2.85)	22.91 (15.37)
Investigations	Blood creatine phosphokinase increased	17	15.52 (9.63–25.01)	15.45 (229.32)	3.95 (2.28)	15.42 (10.34)
Investigations	Transaminases increased	17	14.36 (8.91–23.14)	14.3 (209.86)	3.83 (2.17)	14.27 (9.57)

ROR, reporting odds ratio; PRR, proportional reporting ratio; EBGM, empirical Bayesian geometric mean.

In particular, our study unveiled 36 unexpected and significant disproportionate signals that were not mentioned in pralsetinib’s instructions (**Table 4**). In the investigations, the most intense signals were noted for increased blood calcitonin (n = 5, ROR 793.2, PRR 792.1, IC 9.46, and EBGM 704.2), increased myocardial necrosis marker (n = 21, ROR 202.8, PRR 201.62, IC 7.61, and EBGM 195.44), and bacterial cystitis (n = 4, ROR 137.91, PRR 137.76, IC 7.08, and EBGM 134.85).

**Table 4:** Signal strength of adverse events that are unexpected findings of pralsetinib-related AEs (N = 36) at the preferred term (PT) level that fit four algorithms simultaneously.

System organ class (SOC)	Preferred terms	N	ROR (95% CI)	PRR (χ^2^)	IC (IC025)	EBGM (EBGM05)
Investigations	Blood calcitonin increased	5	793.2 (312.87–2010.93)	792.1 (3511.56)	9.46 (7.74)	704.2 (323.33)
Investigations	Myocardial necrosis marker increased	21	202.8 (131.17–313.53)	201.62 (4063.07)	7.61 (5.94)	195.44 (135.73)
Infections and infestations	Cystitis bacterial	4	137.91 (51.19–371.56)	137.76 (531.5)	7.08 (5.4)	134.85 (58.84)
Reproductive system and breast disorders	Ovarian mass	3	80.96 (25.91–252.96)	80.9 (233.74)	6.32 (4.64)	79.89 (30.8)
Vascular disorders	Labile blood pressure	3	69.95 (22.41–218.34)	69.89 (201.5)	6.11 (4.44)	69.14 (26.67)
Infections and infestations	Fungal foot infection	3	60.21 (19.31–187.78)	60.16 (172.89)	5.9 (4.22)	59.6 (23.0)
Investigations	Carcinoembryonic antigen increased	5	39.12 (16.23–94.3)	39.07 (184.34)	5.28 (3.61)	38.83 (18.6)
Infections and infestations	Pulmonary tuberculosis	6	37.59 (16.84–83.93)	37.53 (212.11)	5.22 (3.55)	37.32 (19.06)
Cardiac disorders	Myocardial injury	3	36.87 (11.85–114.76)	36.84 (104)	5.2 (3.52)	36.63 (14.17)
Infections and infestations	Hepatitis B	5	21.36 (8.87–51.44)	21.34 (96.6)	4.41 (2.74)	21.27 (10.2)
Investigations	Blood phosphorus increased	3	20.5 (6.6–63.71)	20.49 (55.43)	4.35 (2.68)	20.42 (7.91)
Investigations	Protein total decreased	3	19.9 (6.4–61.84)	19.89 (53.64)	4.31 (2.64)	19.83 (7.68)
Metabolism and nutrition disorders	Hyperuricemia	4	17.91 (6.71–47.8)	17.89 (63.6)	4.16 (2.49)	17.84 (7.84)
Infections and infestations	Liver abscess	3	16.59 (5.34–51.53)	16.57 (43.79)	4.05 (2.38)	16.53 (6.4)
Neoplasm benign, malignant, and unspecified (incl cysts and polyps)	Metastatic neoplasm	3	14.82 (4.77–46.02)	14.81 (38.53)	3.88 (2.22)	14.77 (5.72)
Skin and subcutaneous tissue disorders	Onychomadesis	3	13.93 (4.48–43.26)	13.92 (35.89)	3.8 (2.13)	13.89 (5.38)
Investigations	Hemoglobin increased	3	11.71 (3.77–36.36)	11.7 (29.3)	3.55 (1.88)	11.68 (4.53)
Neoplasm benign, malignant, and unspecified (incl cysts and polyps)	Metastases to lymph nodes	4	10.1 (3.79–26.96)	10.09 (32.73)	3.33 (1.67)	10.08 (4.43)
Investigations	Tumor marker increased	4	10 (3.75–26.67)	9.99 (32.3)	3.32 (1.65)	9.97 (4.39)
Respiratory, thoracic, and mediastinal disorders	Nasal dryness	3	9.31 (3–28.91)	9.31 (22.21)	3.22 (1.55)	9.29 (3.6)
Blood and lymphatic system disorders	Myelosuppression	24	8.52 (5.7–12.73)	8.47 (157.93)	3.08 (1.41)	8.46 (6.04)
Infections and infestations	Pyelonephritis	4	8.18 (3.07–21.82)	8.17 (25.15)	3.03 (1.36)	8.16 (3.59)
General disorders and administration site conditions	Sudden death	3	8.07 (2.6–25.06)	8.07 (18.55)	3.01 (1.34)	8.06 (3.12)
Infections and infestations	Bronchopulmonary aspergillosis	4	8 (3–21.33)	7.99 (24.43)	3 (1.33)	7.98 (3.51)
Skin and subcutaneous tissue disorders	Petechiae	4	7.99 (2.99–21.3)	7.98 (24.39)	2.99 (1.33)	7.97 (3.51)
Investigations	Troponin increased	3	7.04 (2.27–21.85)	7.04 (15.52)	2.81 (1.15)	7.03 (2.72)
Neoplasm benign, malignant, and unspecified (incl cysts and polyps)	Metastases to bone	7	6.75 (3.22–14.18)	6.74 (34.2)	2.75 (1.08)	6.74 (3.62)
Gastrointestinal disorders	Esophagitis	3	6.56 (2.11–20.35)	6.55 (14.1)	2.71 (1.04)	6.55 (2.54)
Respiratory, thoracic, and mediastinal disorders	Pulmonary edema	13	5.86 (3.4–10.1)	5.84 (52.13)	2.54 (0.88)	5.84 (3.7)
Psychiatric disorders	Initial insomnia	3	5.72 (1.84–17.75)	5.72 (11.66)	2.51 (0.85)	5.71 (2.21)
Psychiatric disorders	Poor quality sleep	7	5.48 (2.61–11.5)	5.47 (25.55)	2.45 (0.78)	5.46 (2.94)
Gastrointestinal disorders	Toothache	5	5.28 (2.19–12.69)	5.27 (17.29)	2.4 (0.73)	5.27 (2.53)
Neoplasm benign, malignant, and unspecified (incl cysts and polyps)	Metastases to liver	5	4.96 (2.06–11.93)	4.96 (15.78)	2.31 (0.64)	4.95 (2.38)
Musculoskeletal and connective tissue disorders	Muscular weakness	23	4.1 (2.72–6.18)	4.08 (53.53)	2.03 (0.36)	4.08 (2.89)
Gastrointestinal disorders	Dysphagia	18	3.82 (2.41–6.08)	3.81 (37.34)	1.93 (0.26)	3.81 (2.58)
Nervous system disorders	Cognitive disorder	10	3.81 (2.05–7.09)	3.8 (20.65)	1.93 (0.26)	3.8 (2.26)

AEs, adverse events; ROR, reporting odds ratio; PRR, proportional reporting ratio; EBGM, empirical Bayesian geometric mean.

### 3.4 Time to onset analysis of pralsetinib-associated AEs

**Figure 3:**
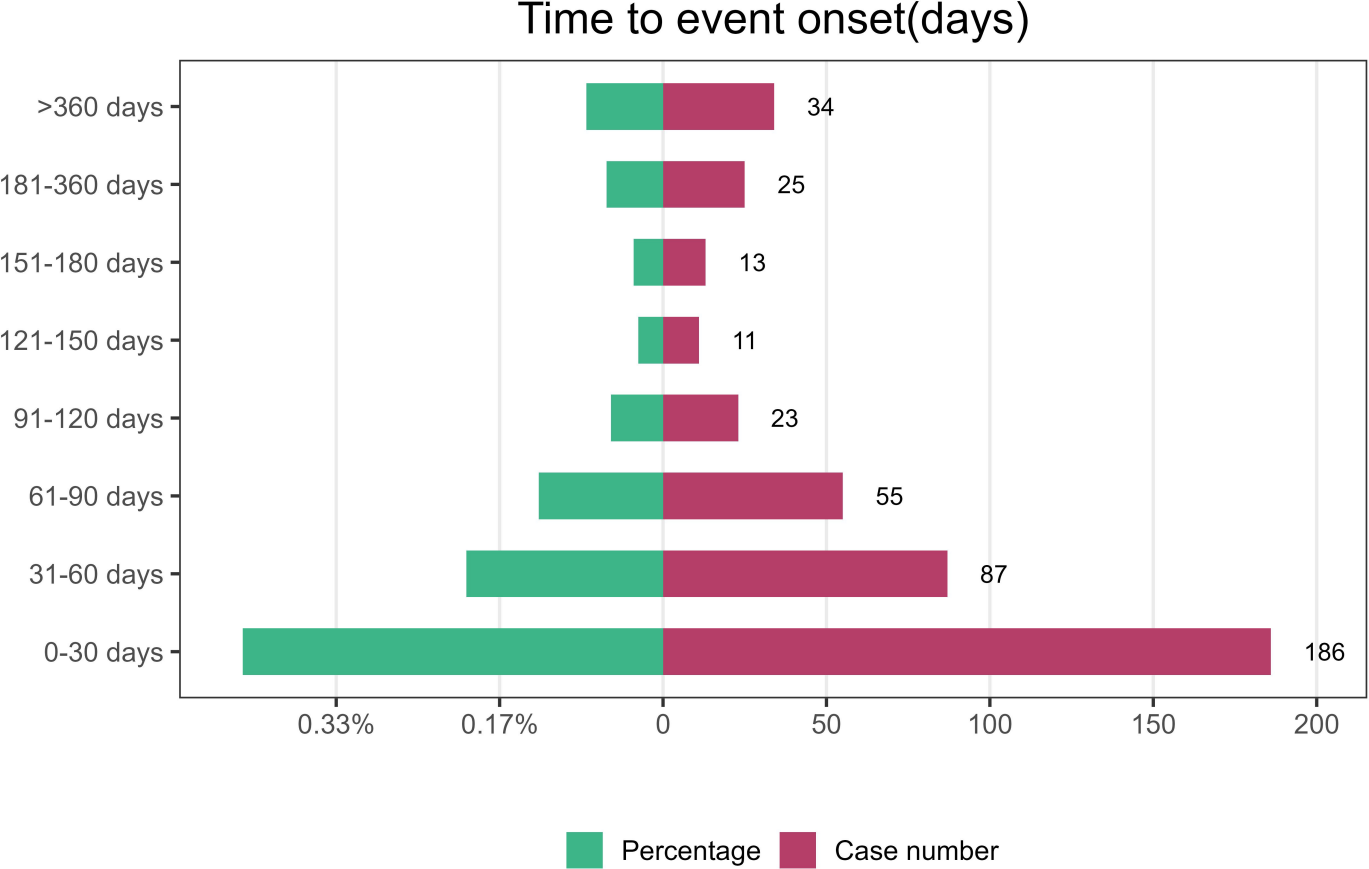
Time to onset of AEs induced by pralsetinib.

The onset times of pralsetinib-related AEs were extracted and analyzed from the FAERS database. The median onset time was 41 days [interquartile range (IQR) 14–86 days]. The majority of the AEs occurred in 30 days (42.86%). Over 3/4 of the AEs occurred in 90 days (75.58%). Notably, data showed that AEs may still occur 360 days after pralsetinib treatment, which accounted for 7.83% of all AEs. The onset times of AEs for each administration of pralsetinib are described in **Figure 3**.

## 4 Discussion

Continuous attention to the risk signals of adverse drug reactions after marketing can help evaluate the safety of drugs and seek the balance of benefits and risks in clinical practice. This study provided a comprehensive description of post-marketing adverse event reports in pralsetinib treatment patients from the FAERS database using four statistical procedures (ROR, PRR, BCPNN, and MGPS).

Our findings show that pralsetinib-associated AEs occur more frequently in women (49.3%) than in men (37.7%). This can be attributed to the main indications of pralsetinib such as thyroid cancer and lung cancer, which are more common in women (11, 12). More than 50% of reporting countries came from the United States, which is mainly related to the earliest launch of pralsetinib in the United States.

In addition, the majority of reports (62.5%) were from consumers rather than healthcare professionals. It suggested that healthcare personnel may be underreporting from medical sources, thus highlighting the need for medical and healthcare personnel to be vigilant when monitoring patients for adverse effects.

Based on the disproportionality analysis, many organs or tissues can be involved. Our study demonstrated that the most commonly significant signals at SOC levels were general disorders and administration site conditions, investigations, and gastrointestinal disorders. Among them, significant AEs mainly included asthenia, increased blood pressure, decreased platelet count, decreased white blood cell count, constipation, and dry mouth, which corresponded with the instruction and clinical trials data (6, 7, 13).

The signals of disproportionality reporting in FAERS showed the most intense risk in the hepatobiliary system. Studies have shown that in cancer patients with non-small cell lung cancer or thyroid cancer treated with pralsetinib, hepatic function abnormality was one of the most common grade 3 or 4 laboratory abnormalities (14). A real-world analysis from the Italian expanded access program indicated that increased liver enzyme levels accounted for 4.9% of grade 3 or worse pralsetinib-related adverse events (15). Our results are in line with the studies regarding the disproportionately high reporting found for hepatobiliary disorders. The instructions also indicate the need to monitor alanine aminotransferase (ALT) and aspartate aminotransferase (AST) prior to initiating pralsetinib, every 2 weeks during the first 3 months and then monthly thereafter and as clinically indicated. Pralsetinib was withheld, the dose was reduced, or pralsetinib was permanently discontinued based on severity.

In this study, we found numerous associations between pralsetinib and systemic adverse events in pharmacovigilance analysis. However, clinical trials cannot provide reliable conclusions due to the strict admission standards, small sample sizes, and short follow-up time when dealing with rare and possible adverse events (5–7, 13). Based on the analysis, in addition to the AEs commonly in the instruction, we focus on the signals classified as strong signals in the pralsetinib-associated AE reports (**Table 4**). Increased blood calcitonin has the strongest signal, but studies have shown that calcitonin levels are elevated in patients with medullary thyroid cancer (16, 17); therefore, the adverse event of increased blood calcitonin may be related to the disease condition and cannot be attributed to taking pralsetinib. The signal of pralsetinib in metastases to lymph nodes (n = 4), bone (n = 7), and liver (n = 5) may be due to the failure of one prior therapy, and patients may still have tumor metastasis and tumor progression when the efficacy of pralsetinib is imperfect. In addition, owing to the characteristics of local invasion and distant metastasis of malignant tumors, it may be illogical to estimate whether tumor metastasis is caused by pralsetinib only by adverse drug reaction (ADR) signals.

It is worth noting that these high AE signals, such as increased myocardial necrosis marker (n = 21), myocardial injury (n = 3), and increased troponin (n = 3), suggest that pralsetinib may present a risk of cardiotoxicity. In recent years, prolonged QT interval has been reported during pralsetinib treatment in clinical practices (7, 18, 19). Selpercatinib, also a selective inhibitor of RET, has been shown to have an adverse effect of prolonged QT interval in the drug label. In this situation, it is important to conduct more studies to evaluate the long-term safety and efficacy of pralsetinib. It is recommended to add cardiac function monitoring measures in clinical trials to better assess the safety of pralsetinib. It is hoped that our study can provide insights into the clinical application of pralsetinib. At the same time, considering the risk of life-threatening torsional tachyarrhythmia induced by QT prolongation, clinicians should exercise caution to avoid administering medications that prolong the QT interval.

PTs with significant signals were observed for bacterial cystitis, fungal foot infection, pulmonary tuberculosis, hepatitis B, liver abscess, pyelonephritis, and bronchopulmonary aspergillosis among the SOC level of infections and infestations. A single-center study from Korea showed two adverse events of extrapulmonary tuberculosis in patients with RET fusion-positive non-small cell lung cancer treated with pralsetinib (20). It is well established that for autoimmune diseases or cancers, JAK inhibitors are associated with an increased frequency of infection (21–23). Pralsetinib may therefore predispose patients to infections as a result of their off-target effects on JAK1/2 (24). In addition to pneumonia, recurrent and serious infectious adverse events appear to be of concern under the pralsetinib therapy. Increasing awareness of these infections is relevant clinically because the associated lesions can be misdiagnosed as disease progression. Early detection and timely application of anti-infective drugs are key to treatment. Further studies are needed to provide proper guidelines to physicians for managing infectious adverse events under pralsetinib therapy.

Additionally, AEs in the reproductive and breast system should be of concern. Notably, ovarian mass (n = 3, ROR 80.96, PRR 80.9, IC 6.32, and EBGM 79.89) was found in our study. Considering the strong disproportionation signal, it suggested that doctors should closely monitor the potential adverse effects during treatment in this specific population of women.

Psychiatric AEs should be distinguished from other causes. Notably, we found disproportionality reporting, such as poor quality sleep (n = 7) and initial insomnia (n = 3). At least 30% of patients report in fact psychosocial distress and mental disorders, and even a higher percentage report unrecognized psychosocial needs or untreated psychosocial disorders as a consequence of cancer at some point during the cancer trajectory (25). Psychosocial intervention can improve psychological and physiological adaptation indicators in cancer patients (26). Therefore, appropriate psychological intervention is necessary.

The results of our study indicated that the median onset time was 41 days, and most of the cases occurred within the first 1 month (n = 186, 42.86%), 2 months (n = 87, 20.05%), and 3 months (n = 55, 12.67%) after pralsetinib treatment. Most of the AEs occurred within the first 3 months. It suggested that we should be vigilant about the AEs associated with pralsetinib in the first month. Early recognition of AEs caused by pralsetinib therapy is important, as these adverse drug effects can be life-threatening.

Although the current study showed a potentially insightful relationship between the use of pralsetinib and the odds of reporting AEs in the FAERS, it is not without limitations. FAERS database is a self-reporting system, which may have some limitations such as missing reports, duplicate reports, or incomplete case information. In addition, the reported information in this database lacks the underlying disease and drug combination of patients, so it may have a certain impact on the mining results of adverse reaction signals. This study confirmed that pralsetinib was associated with some adverse reactions; however, it cannot represent the population incidence. The signal of adverse events only illustrates a statistical association; further clinical observation and prospective studies are needed to determine whether a biological causation exists.

## 5 Conclusion

Our pharmacovigilance analysis of real-world data from the FEARS database revealed the safety signals and potential risks of pralsetinib usage. These results can provide valuable evidence for further clinical application of pralsetinib and are important in enhancing clinical medication safety.

## Data Availability

The original contributions presented in the study are included in the article/**Supplementary Material**. Further inquiries can be directed to the corresponding authors.
